# The effect of COVID-19 pandemic and wearing face masks on ophthalmology practice: What is known so far? A narrative review

**DOI:** 10.3389/fmed.2022.1019434

**Published:** 2022-11-28

**Authors:** Luai Abu-Ismail, Khayry Al-Shami, Manar Al-Shami, Abdulqadir J. Nashwan

**Affiliations:** ^1^Department of Ophthalmology, Islamic Hospital, Amman, Jordan; ^2^Department of Clinical Medical Sciences, Faculty of Medicine, Yarmouk University, Irbid, Jordan; ^3^Princess Basma Hospital, Ministry of Health, Irbid, Jordan; ^4^Hamad Medical Corporation, Doha, Qatar

**Keywords:** ophthalmology, face mask, COVID-19, ocular, eye

## Abstract

Face masks, along with other preventive measures, can help slow the spread of COVID-19. Despite the positive effect of the mask in combating the virus, it has some negative effects on the human body that must be followed up on and reduced. In this study, we discuss the impact of wearing face masks on the eye and the common issues associated with using them. The literature search was conducted using electronic databases such as PubMed and Google Scholar. Only articles published in English were included. A total of 39 relevant articles were deemed eligible. After the duplicate articles were removed, the titles and abstracts of 20 papers underwent full-text screening. The review comprised both prospective and retrospective investigations, case reports, and a series of reporting ocular symptoms following the use of face masks. The COVID-19 pandemic affected ophthalmology practices in managing patients. New factors must be considered, especially when dealing with anti-VEGF injections, such as the risk of endophthalmitis, tests and symptoms of patients with glaucoma, and the emerging symptoms associated with the COVID-19 vaccination. The use of face masks and breathing aids seemed to influence the tear film.

## Introduction

In the past 2 years, the world has witnessed the advent of the largest outbreak and health crisis since World War II, which is the COVID-19 pandemic caused by the severe acute respiratory syndrome coronavirus 2 (SARS-CoV-2), which has dramatically affected the global healthcare system and different aspects of life due to its rapid spread among people and the variation of symptoms noticed from one patient to another. This has changed many principles of healthcare practices, especially in ophthalmology. Recently, the World Health Organization issued a guiding protocol on the use of masks and, therefore, to reduce the spread of disease (1). Face masks became a very important factor in controlling the pandemic, along with other controlling measures (2). Wearing a mask helps reduce the spread of COVID-19 significantly as it covers two parts of the T-zone, namely, the nose and the mouth, while the eye area remains uncovered, which makes the person vulnerable to receiving or transmitting COVID-19. Moreover, different ocular manifestations associated with SARS-CoV-2 in the anterior and posterior segments of the eye were noted (3). The virus was detected in tears and conjunctival samples, implicating the eye as a potential route for viral entry (4, 5). Central retinal vein occlusion (CRVO) and central retinal artery occlusion (CRAO) were two of the many vascular manifestations of COVID-19 (3, 4). In this review, we assessed the effect of the strict regulations of wearing masks on the practices of different ophthalmic procedures, investigations, and treatments, along with the patient's eye health.

## Anti-VEGF injection complications

During the pandemic, since 2020, concerns were raised regarding the possible effects of using face masks on eye health and ophthalmology practices all over the world. During a virtual meeting on March 2020, a 14-member Vision Academy Steering Committee debated the key challenges of managing patients receiving injections during the COVID-19 pandemic (6). During that time, several international organizations changed their guidelines for ophthalmologists in dealing with patients accordingly. In the literature, few articles were published considering the safety of face mask use while administering intravitreal treatment. Infectious endophthalmitis is the most feared complication of intravitreal injections (7). A recent U.S. retrospective study over 5 years reported an endophthalmitis rate of 0.036% (1: 2,778) (8). Although the rate is low, it is still a major concern after the procedure. Trying to understand the source of infection was the main area of interest to prevent postinjection complications. In the 2018 European Society of Retina Specialists' expert consensus recommendations for the use of surgical face masks or a no-talking policy during the injection (9), wearing a face mask was an important measure to control the pandemic in December 2020, which was recommended by the WHO (10). Many endophthalmitis cases may be caused by salivary flora contaminating the operative field through droplet spread or aerosolization (11, 12). Even though the source of the droplets is not clear, it should be considered if we intend to reduce the possibility of this problem and have a better outcome that follows the pandemic restrictions and measures. Therefore, proper use and fit of face masks are important, as they might be a possible risk factor during the procedure when they are worn by patients these days because of the pandemic. The first experimental study that tried to develop a better understanding of this issue was published in June 2020 (7). It involved 10 patients using three different types of face masks monitored by two professional cameras, with 90 trials recorded. Air leaks were found in every type of mask that was investigated. In 81% (73/90) of cases, air jets emanating from the mask's top edges were seen pointing toward the eyes. To explain how infections spread, Carl Flügge was the first to propose the droplet theory. Mikulicz was the first to recommend the use of face masks to stop the spread of germs from medical professionals' mouths during surgery in 1897 (13, 14). Wen et al. (15) demonstrated that oral bacterial spread reduced significantly during a simulated intravitreal injection when healthcare providers used surgical face masks or remained silent. A meta-analysis concluded that there should be strategies to minimize oropharyngeal droplet transmission, which may include wearing surgical masks as streptococcal isolates were approximately three times more frequent after intravitreal anti-VEGF injection than after intraocular surgery (11). Streptococcus species are thought to contaminate operative fields by aerosolization or droplet spread (16–20). Applying medical adhesive tape across the upper border of the face mask was recommended to prevent air leaks. New literature that was published in 2021 discussed this issue, which was only discussed in five articles published during that year. In two large retrospective comparative cohort studies, it was concluded that the wearing of face masks by the patients and doctors during the procedure did not influence the rate of postinjection endophthalmitis, but the authors noted that the cases associated with positive cultures of oral flora were decreased (21, 22). It was clear that taping face masks reduced the quantity of air particles directed toward the eye during the procedure, which suggests a reduction in bacterial dispersion (23–25).

## Glaucoma and standard automated perimetry test (SAP)

In patients with glaucoma, the effects of the COVID-19 pandemic on the aspects of eye carecan cause problems and have negative results on the accuracy of patient follow-up. Due to COVID-19 measures, patients have been forced to wear face masks, which has resulted in reduced accuracy of visual field examinations or measurement tests, especially if the face masks are not properly sealed (26, 27). In a study on patients with glaucoma, in which all patients who underwent SAP from May to October 2020 were enrolled, the SAP test was performed again for the enrolled patients after wearing the mask to observe the changes in their visual field since the beginning of the COVID-19 pandemic. The study included 127 patients who were divided into two groups as follows: those who wore surgical face masks (101 patients) and those who wore cloth face masks (26 patients). The results were as follows: low reliability of SAP appeared in 23 patients of the whole sample, and lower visual field defects were observed in three patients of the whole sample. The percentage of low reliability of SAP in people who wear cloth face masks is five times higher than that of people who wear surgical face masks. We conclude that unsuitable face masks may cause visual field defects, such as increased severity of glaucoma or decreased test reliability. Gluing the top edges of face masks is a good way to prevent problems with the field of vision and get a good test result (28).

In a study whose objective was to look at how the COVID-19 epidemic affected glaucoma surgical procedures in the United Kingdom, they found that trabeculectomy was the procedure of choice for 61 (87%) glaucoma specialists. Before the COVID-19 pandemic, 51 (73%) of the respondents reported performing minimally invasive glaucoma surgery (MIGS) procedures. The most commonly performed MIGS procedure was the iStent Inject (51%), followed by XEN 45 (36%) and Preserflo (17%). Following the onset of the COVID-19 pandemic, 43 (61%) respondents reported modifying their glaucoma surgery practice. Of the glaucoma specialists who modified their surgical practices, 21 (43%) specifically reduced the number of trabeculectomy procedures performed. In combination, diode laser therapy (both micropulse and conventional trans-scleral cyclodiode) was the most common alternative procedure. Glaucoma drainage devices, deep sclerectomy, and Preserflo were also commonly chosen alternatives. Table 1 clarifies the results of the study (29).

**Table 1:** Patterns of change in glaucoma surgery practice according to consultant experience (29).

**Consultant experience (years)**	**Changed glaucoma surgery practice (%)**	**Restricted/ reduced trabeculectomy (%)**
0–5	7 (31)	8 (36)
6–10	11 (53)	10 (48)
11–15	2 (27)	1 (12)
16–20	4 (42)	3 (31)
>20	4 (29)	3 (21)

## Respiratory support devices

A continuous positive airway pressure (CPAP) machine is a form of positive airway pressure breathing machine that applies moderate air pressure continuously. It keeps the airways open constantly in people who can breathe on their own but need help keeping the airways unobstructed. It is an alternative to a positive end-expiratory pressure (PEEP) device; both methods open the alveoli of the lungs, allowing more surface area for ventilation. A positive end-expiratory pressure device applies positive pressure only at the end of exhalation, while a CPAP device applies continuous positive pressure during the breathing cycle. Therefore, while the CPAP device is working, the ventilator itself does not operate, does not provide additional pressure above the level of the previous device, and requires patients to start all their breaths when using it compared to the ventilator (30). In a case report, while following up on the condition of a 48-year-old man with sleep apnea, the patient indicated that his condition had improved and that using CPAP made him feel comfortable, but he also had an unusual side effect, that is, he felt his left eyelid would explode when he opened his eyes; he felt that the air was escaping from it. Therefore, the patient tried the APAP (automatic positive airway pressure) or its other name, the total face mask, and he felt much better than with CPAP. In conclusion, the return of air to the eye is a rare complication of CPAP therapy that may be more common in patients with damaged anatomical structures of the lacrimal duct. Several interventions have been attempted to overcome these complications. In our case, the use of a full-face mask is an effective and well-tolerated new solution (31).

## Contact lenses and using masks

Contact lenses became one of the most popular devices used for cosmetic and medical issues. It has been proven that using contact lenses without proper hand hygiene and careful care for the eye's health may result in putting the eye at higher risk of infection with pathogenic bacteria added to its effect on the cornea and the eye's health (32). As we explained previously, wearing a mask negatively affects the tear film as it weakens it and makes the eye vulnerable to dryness and eye surface diseases. The use of the mask by people who use contact lenses leads to a doubling of the speed of eye dryness and an increase in the possibility of infection with eye surface diseases. In other words, faster evaporation of the lacrimal membrane results in dry patches on the surface of the eye, irritation, and discomfort (33). In another study, a questionnaire that consisted of nine questions related to the eye condition of contact lens users when wearing a mask was developed. Several questionnaires were used to make this questionnaire, such as OSDI, DEQS, UNC DEMS, NEI VFQ 25, SPEED, DEQ-5, DEEP, and CLDEQ-8. The questions were formulated to obtain information related to demographic data such as gender, age, occupation, type of contact lens, and when to replace it. In addition, contact lens conditions were paid attention before and after the epidemic. The epidemic affected the use of contact lenses due to the frequency of mask use, and some eye symptoms were associated with wearing contact lenses with a mask (34, 35). The study included 177 people with an average age of 38.39 ± 10.9 years, as it appeared that 35% of the whole sample had allergies. People who replace contact lenses were divided into three sections as follows: one that replaced lenses every month, which made up 61.7% of the allergic sample; some who replaced them every 2 weeks, which amounted to 8.5% of the allergic sample; and some who replaced them daily, which amounted to 28.8%of the allergic sample. From the results of the questionnaire, we observed that there was also a significant decrease in the use of contact lenses compared to the time before the pandemic (33).

Symptoms related to dry eyes were present in 61.5% of the participants. Around 81% of those who had ocular symptoms did not report any change in the severity of their symptoms by wearing the mask, while 17.5% had their symptoms worsened, and 1.2% had their symptoms improved with the use of contact lenses [32].

Wearing the N95 mask was not limited to the COVID-19 pandemic; in 2002, in China, specifically in Guangdong, an outbreak of atypical pneumonia was found, especially among healthcare workers and their families, where healthcare workers were assigned to wear masks and protection when dealing with people suspected of having SARS (36, 37). The N95 mask is so named because it protects against respiratory droplets; the letter “N” stands for “not oil-resistant,” and the number “95” indicates that the mask is 95% effective at filtering particles with an average diameter >0.3 m^2^ (38). After a period of using the mask, some health workers felt headaches resulting from the prolonged use of the mask. In a study involving 212 health workers, 79 reported having headaches associated with wearing masks, 23 said that the headache recurred six times a month, six people took very long vacations, and 47 took painkillers for headaches. The study concluded that wearing the mask leads to a headache and that the tension and recurrence of headaches decrease as the time spent wearing the mask decreases (39). Wearing the mask not only leads to hemodynamic changes, but its effect extends to the choroidal and retinal blood circulation as a result of carbon dioxide retention, which has a vasodilating effect (40). In another study conducted on health sector workers, the prolonged use of the mask led to increased inhalation of carbon dioxide gas, which leads to the occurrence of strikes, changes in the choroidal circulation, and increases in the choroidal thickness (41).

## Vision and falling down

Masks constantly block areas of lower peripheral vision, even for those who do not wear glasses, in addition to wearing glasses that impair vision (fogging glasses). For spotting and avoiding any threats in the area as well as securely arranging our steps, visual information from the lower outside field is crucial. The likelihood of using this crucial sensory information when walking is decreased when a mask is worn, which may raise the risk of tripping or falling (42–44). It seems logical to argue that, when wearing masks, people always glance down at their feet. They will receive the visual data that they would typically acquire while looking ahead with their lower peripheral vision. Such a plan has already started to take shape. Although it seems contradictory, we would contend that this idea is erroneous. Considering the two purposes of vision when walking will help you understand why (45). First, vision is employed to identify hazards and designate secure walking routes, particularly for the elderly. Planning is frequently challenging when bending your head. According to recent research employing eye-tracking equipment, older people make more errors when they gaze at their feet than when they look ahead and consider the possibility of tripping (46, 47). Second, maintaining balance involves a combination of additional sensory input and visual information, particularly from the periphery. This is improved by minimizing head and eye movement during walking to offer a stable visual “anchor” as the primary sensory input for adjusting the balance. It can even cause severe instability as it requires frequent and large movements of the head and eyes, which can cause an imbalance between vision and vestibular reflexes (48, 49). In short, advice to simply “appear down” even as carrying masks can sarcastically compromise stability, interfering with fine-tuned structures that use imagination and prescience to offer protection even as walking. This impacts now not only the elderly but also anybody whose stability is especially dependent on imagination and prescience, that is, individuals with Parkinson‘s disease or diabetic sensory neuropathy (50). It is essential to make sure that the mask fits snugly around the nostrils and cheeks. Good health not only minimizes the risk of COVID-19 infection but also reduces visible damage and minimizes eyeglass fogging. In the future, it may be possible to create bespoke masks for the ultimate in shape and health with minimal impact on vision and comfort. In the meantime, those who wear glasses can use anti-fog technology like swimmers (51). Threatened organizations must be advised to “take time” rather than “get off.” Walk slowly before you start walking. This gives you plenty of time to explore nearby boundaries and plan a safe route (52). Slowing down also reduces the desire for large, rapid movements of the head and eyes when walking. Slow walking speed has disadvantages in addition to large fluctuations in speed and decreased physical activity. Slowing down may not be the most volatile strategy given that you risk doing extra searches and do not currently make adjustments. Wearing a mask is essential during the COVID-19 pandemic, especially when you are with the elderly in a high-risk environment. It is vital to reduce the performance impact on gait protection to maximize the use of masks and reduce the likelihood of leaving sports that require masks. Future research is needed to evaluate a variety of protection technologies, including slowing down the use of recommendations and explicit masks to enable evidence-based, fully open fitness recommendations (53).

## Face mask and tear film

The eye has a mechanism to maintain its moisture and protect it from dust and dirt, which is the tear film. The tear film consists of three layers, arranged from outside to inside, as follows: from the outside, the fatty layer; in the middle, the aqueous layer; and from the inside, the mucous layer. These three layers that make up the tear film are made up of lacrimal glands, accessory lacrimal glands, goblet cells, and the meibomian glands (54, 55). Any injury that affects the tear film leads to direct exposure of the eye to air and dust, which leads to dry eyes. In contrast, there are some physiological factors such as aging and menopause and pathological factors such as Sjögren's syndrome, lacrimation deficiency, meibomian gland deficiency, diabetes, adenomyosis, and Hashimoto's thyroid disease; environmental factors such as prolonged screen time, air conditioning, and smokers; and iatrogenic factors such as contact lenses, medications, eye surgery, and wearing masks (56, 57). The current problem that we are discussing is the effect of face masks, as wearing them in this pandemic has become mandatory because of their significant impact in limiting the spread of the disease, but at the same time, wearing them for long periods of time increases the chances of dry eyes, as a cross-sectional study was conducted on a group of patients to measure the changes in the tear film and the susceptibility to ocular surface diseases. It was found clearly that people who wear a face mask for a short period are less susceptible to diseases of the eye surface, in addition to the fact that the tear film is not affected to a great extent in contrast to people who wear a face mask for long periods (58, 59). Another study reported that the use of surgical masks throughout the day leads to a significant reduction in NI-BUT, regardless of age, gender, or OSDI score, which should raise the need to consider the prolonged use of surgical masks as a risk factor for evaporative dry eye disease (60). However, a study performed in Jordan showed no relation between wearing masks and dry eye disease, which makes it clear that more studies need to be conducted to investigate this issue (61).

**Figure 1:**
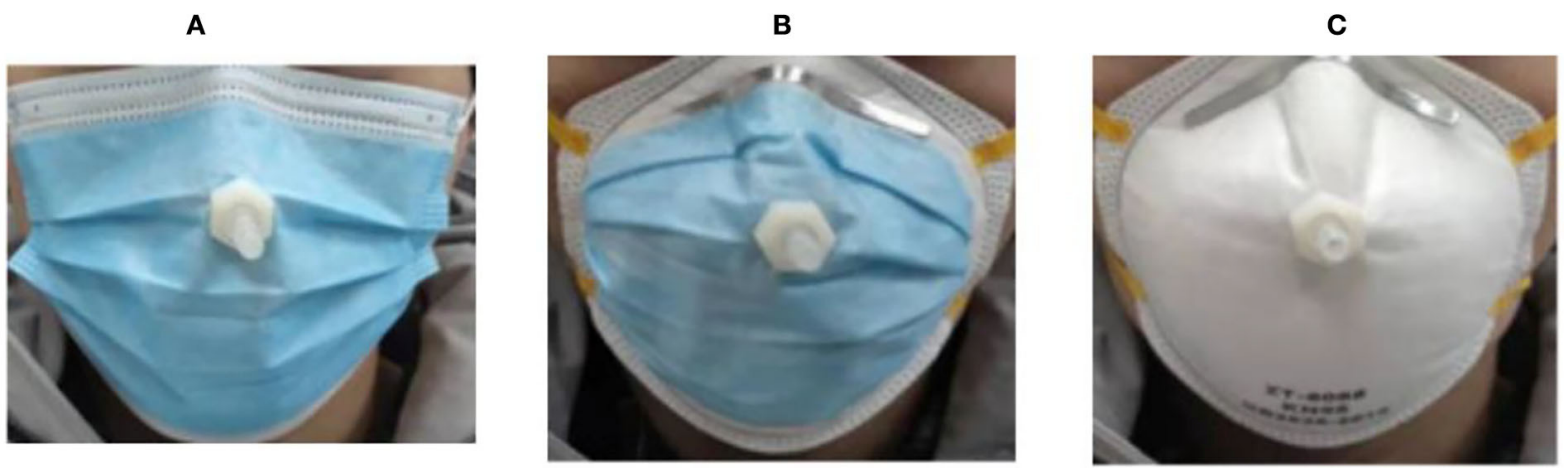
Three types of face masks: **(A)** modified face mask, **(B)** N95 face mask, and **(C)** surgical face mask (62).

In a study that compared three types of masks in terms of protection and comfort in use (refer to Figure 1), the authors used gas chromatography to test the N_2_, CO_2_, and O_2_ concentrations inside the three face masks. The three masks had N_2_ concentrations of ~76%, which was lower than the 78% N_2_ content in the environment. This occurred because of the exhaled air's high water vapor content, which forced the N_2_ out of the body and decreased its percentage in the atmosphere. The modified mask had an O_2_ concentration that was higher than the N95 mask but lower than the surgical face mask. Thus, it was not surprising that the modified face mask's CO_2_ content was higher than that of the surgical mask but lower than that of the N95 mask. The results shown in Table 2 are in line with those from the earlier continuous monitoring (62).

**Table 2:** CO_2_, O_2_, and N_2_ concentrations inside three face masks measured by gas chromatography (62).

	**CO_2_ (%)**	**O_2_ (%)**	**N_2_ (%)**
Surgical	1.64	18.81	76.96
Modified	2.22	18.05	76.89
N95	2.71	17.08	76.58

## Ophthalmic manifestation of the COVID-19 vaccine

As mentioned earlier, the COVID-19 pandemic has greatly affected the world in general and the medical sector in particular. It has caused the death of more than 3 million people worldwide, which necessitated the world to intervene quickly to produce a vaccine to fight this pandemic. Since the discovery of the disease in 2019, many vaccines have been developed to fight the pandemic. We also know that every useful thing has a bad side, as there are some general side effects of using COVID-19 vaccines, and among the most important of these symptoms are those related to the eye, so we will discuss why the eye has sensitivity and its impact on human life (63, 64). According to the phenomenon of antibody-dependent enhancement (ADE), the COVID-19 vaccine can affect the eye or the eye nerves through autoimmunity against the eye structure, as this immune phenomenon leads to inflammation of the retina, the choroid, the optic nerve, and the uvea (65). Some authors reported panuveitis with thickening of the choroid in conjunction with the anterior chamber and vitreous inflammation, as well as anterior uveitis, after a dose of the COVID-19 vaccine. An effect on the retina and optic nerve was also noted. One of the most prominent vaccines found to affect the eye is the AstraZeneca vaccine, whose use led to the emergence of acute central serous retinopathy. There is also the Pfizer-BioNTech vaccine, the use of which led to the occurrence of acute macular retinopathy, the emergence of Bell's palsy, retrograde orbital conjunctivitis, and severe visual impairment and visual field defects. In other studies, there was a bilateral superior ophthalmic vein thrombosis after the use of the ChAdOx1 nCoV-19 vaccine (66–68).

## Conclusion

In conclusion, wearing face masks during the pandemic was the major controlling factor. Although it has many benefits regarding the prevention and control of various infectious diseases, it had an impact on the ophthalmology patient's health specifically. Although it appeared to have a positive influence on controlling the risks of endophthalmitis to some point, it has the opposite impact on the test reliability of patients with glaucoma and the incidence of dry eye disease-related issues, especially among ICU patients. The major limitation of our study was the limited number of articles published so far. The effect of face mask regulations in the ophthalmology field needs further studies to develop a better understanding of its effect on the different areas of investigations and eye diseases.

## Author contributions

LA-I, KA-S, MA-S, and AN: data collection, literature search, and manuscript preparation. All authors read and approved the final manuscript.

## Conflict of interest

Author AN was employed by Hamad Medical Corporation. The remaining authors declare that the research was conducted in the absence of any commercial or financial relationships that could be construed as a potential conflict of interest.

## Publisher's note

All claims expressed in this article are solely those of the authors and do not necessarily represent those of their affiliated organizations, or those of the publisher, the editors and the reviewers. Any product that may be evaluated in this article, or claim that may be made by its manufacturer, is not guaranteed or endorsed by the publisher.
